# *DeepRes*: a new deep-learning- and aspect-based local resolution method for electron-microscopy maps

**DOI:** 10.1107/S2052252519011692

**Published:** 2019-09-18

**Authors:** Erney Ramírez-Aportela, Javier Mota, Pablo Conesa, Jose Maria Carazo, Carlos Oscar S. Sorzano

**Affiliations:** a Biocomputing Unit, National Center for Biotechnology (CSIC), Calle Darwin 3, Campus Universidad Autónoma de Madrid, Cantoblanco, 28049 Madrid, Spain; b Universidad CEU San Pablo, Campus Urbanizacion Montepríncipe, Boadilla del Monte, 28668 Madrid, Spain

**Keywords:** *DeepRes*, electron microscopy, single-particle analysis, local resolution, 3D reconstruction and image processing, single-particle cryoEM, structure determination, cryo-electron microscopy

## Abstract

In this article, a method is presented to estimate a new local quality measure for 3D cryoEM maps that adopts the form of a ‘local resolution’ type of information. The algorithm (*DeepRes*) is based on deep-learning 3D feature detection. *DeepRes* is fully automatic and parameter-free, and avoids the issues of most current methods, such as their insensitivity to enhancements owing to *B*-factor sharpening (unless the 3D mask is changed), among others, which is an issue that has been virtually neglected in the cryoEM field until now. In this way, *DeepRes* can be applied to any map, detecting subtle changes in local quality after applying enhancement processes such as isotropic filters or substantially more complex procedures, such as model-based local sharpening, non-model-based methods or denoising, that may be very difficult to follow using current methods. It performs as a human observer expects. The comparison with traditional local resolution indicators is also addressed.

## Abstract 2

A method (*DeepRes*) is presented to estimate a new local quality measure for 3D cryoEM maps that adopts the form of a ‘local resolution’ type of information. *DeepRes* is fully automatic and parameter-free and avoids the issues of most current methods, such as their insensitivity to enhancements owing to *B*-factor sharpening, among others.

## 1. Introduction   

Single-particle cryo-electron microscopy (cryoEM) has become a powerful technique for the three-dimensional (3D) structure determination of biological molecules. Recently, advances in instrumentation and software have dramatically improved the potential of single-particle cryoEM, generating density maps with a high level of detail. The quality of the map thus obtained is usually evaluated in terms of resolution. Different measures have been proposed to determine map resolution in cryoEM (Sorzano *et al.*, 2017 ▸). Currently, the most-used definition is based on the Fourier shell correlation (FSC) curve (Saxton & Baumeister, 1982 ▸; Saxton, 1978 ▸; Harauz & van Heel, 1986 ▸). However, resolution is not a concept that can be uniquely defined. From the point of view of the microscope, we may define the resolution based on optics considerations (for instance, the Rayleigh criterion determines the minimum spatial separation between two points so that the two points can still be separated). From the point of view of signal processing, we may define the resolution in terms of some form of signal and noise comparison [for instance, the frequency at which there is more signal than noise; this is used in both the *ResMap* (Kucukelbir *et al.*, 2014 ▸) and *MonoRes* (Vilas *et al.*, 2018 ▸) approaches]. From the algorithmic reproducibility point of view, we may define the resolution as the maximum frequency at which the correlation between two bandpass-filtered versions of two reconstructions performed with the same algorithm but applied to two independent data sets is above a given threshold (this is the definition of the Fourier shell correlation). Or, from the point of view of the nature of the objects being visualized, for instance, we may define the resolution as the maximum frequency at which the features in the reconstructed object are consistent with the features observed in biological macromolecules filtered at that frequency. All of these definitions make sense and they all assess the quality of the reconstruction by attending to different aspects of the problem. In addition to this ambiguity, all of the methods described above require the choice of a threshold for the measured quantity (cross-correlation, signal-to-noise ratio *etc.*), which has also caused long debates on the issue. Additionally, it is already well known that the quality of a reconstruction depends on the region of the macromolecule (some regions are better resolved than others; Cardone *et al.*, 2013 ▸; Kucukelbir *et al.*, 2014 ▸; Vilas *et al.*, 2018 ▸) and even on the direction (some directions are better resolved than others owing to an uneven angular distribution; Sorzano *et al.*, 2001 ▸; Unser *et al.*, 2005 ▸), although we will not consider directional effects in this work.

One of the first methods for considering the local characteristics of the map for calculation of the resolution was *BlocRes* (Cardone *et al.*, 2013 ▸). This method calculates the resolution based on the FSC but using a moving window on the maps. In addition to having the limitations that are inherent to use of the FSC (focused on reproducibility only, lack of sensitivity to isotropic, nonvanishing filters *etc.*), *BlocRes* incorporates the variable of the window size.

The most-used method to date for local resolution estimation is *ResMap* (Kucukelbir *et al.*, 2014 ▸), which is based on the detection of a sinusoidal wave above the noise level for each point on the map. Based on a similar principle, of detecting energy at different frequencies above noise, but on a totally different signal-processing approach, *MonoRes* (Vilas *et al.*, 2018 ▸) was proposed. *MonoRes* is based on the use of monogenic signals, extracting the monogenic amplitude at different frequencies and comparing it with the monogenic amplitude of the noise at the corresponding frequency; a directional local resolution extension of *MonoRes* has also been proposed (manuscript under review). The main limitation of these two latter methods is that they require an estimate of the noise variance.

On the other hand, it is very common to introduce some form of map enhancement, from global *B*-factor correction to more complex post-processing operations such as model-based or non-model-based sharpening [as in *LocScale* (Jakobi *et al.*, 2017 ▸) and *LocalDeblur* (Ramírez-Aportela *et al.*, 2019 ▸), respectively] or the introduction of nonlinear noise-suppression operations, as in *Xmipp Highres* (Sorzano *et al.*, 2018 ▸). In all of these cases, our current resolution indicators lose effectivity: in the former case (*B*-factor correction) because they are insensitive to this operation (see the supporting information and Supplementary Fig. S1) and in the latter case because the estimation of the level of noise in the map is also affected by these operations, impacting on the very basic mathematical framework on which they are based (operationally; if still used in this way this weakness translates into an overestimation of resolution).

However, it is certainly non-intuitive that our current resolution estimations are insensitive to operations that are aimed to increase the quality of our maps, even if sometimes these enhancements are mostly targeted to help the modeling task. Indeed, visibly different maps can present the same resolution as estimated by methods based either on the FSC or on some form of signal-to-noise ratio (SNR; see the supporting information and Supplementary Fig. S1). Therefore, we wanted to derive another ‘local quality measure’, formally of the type of a local resolution, that could be used in these cases and that indeed could ‘follow’ the results of these enhancing operations (*i.e.* it produces a better value when the map is supposed to be better by the application of some post-processing). Therefore, our primary motivation in developing *DeepRes* was to reconcile ‘what you see’ when working with a map with ‘what you get’ in terms of the calculated value of local resolution. Note that currently two maps, one sharpened and the other unsharpened, may have the same local resolution estimation but are certainly not judged as being the same by a human observer. An additional, more technical, motivation for our work on *DeepRes* was to have a way to calculate local resolution in those situations in which we did not have a proper noise estimation (as could be the case, for instance, when the reconstruction algorithm incorporates some noise-suppression prior, as in *Xmipp Highres*; Sorzano *et al.*, 2018 ▸).

Naturally, a logical question for the cryoEM practioner is ‘and which is the true, final, local resolution?’. We will present our work in this area, which clearly indicates that some general consensus can indeed be achieved, even when working with very different ‘definitions’ of what resolution is.

In brief, in this work we introduce a new algorithm (*DeepRes*) for measuring the local resolution of biological macromolecules reconstructed by cryoEM. It is based on the comparison of the features of biological macromolecules observed at a particular resolution with the features observed in the map under evaluation. Our proposal makes use of deep learning. Deep learning is a new area of artificial intelligence that has recently emerged (Bengio, 2009 ▸) and that has already been successfully applied in cryoEM with excellent results (Wagner *et al.*, 2018 ▸; Su *et al.*, 2018 ▸). Avramov and coworkers have demonstrated that deep-learning models can learn resolution patterns from cryoEM density maps (Avramov *et al.*, 2019 ▸). Our newly proposed method, *DeepRes*, overcomes some of the limitations of current local resolution methods such as their counter-intuitive insensitivity to isotropic, non­vanishing filters (*B*-factor correction) and many sharpening algorithms (for example *LocalDeblur*), and their inability to estimate the resolution when there is no region to estimate the noise distribution (a single masked reconstruction) or when the reconstruction method strongly suppresses noise in the reconstruction (for example *Xmipp Highres*; Sorzano *et al.*, 2018 ▸).

## 2. Methods   

The *DeepRes* algorithm is based on a convolutional neural network (CNN). The idea of our method is ‘to teach’ a neural network the characteristics of density maps filtered at different frequencies, creating a general network that can be used to estimate the local resolution of cryoEM maps.

### 2.1. Training data set   

A set of 15 000 3D nonredundant macromolecule structures (including proteins and nucleic acids; Levy *et al.*, 2006 ▸) was selected for the training of our 3D convolutional neural networks (CNNs). Each atomic model was simulated as a 3D density map, calling the function *xmipp_volume_from_pdb* from the *Xmipp* package (de la Rosa-Trevín *et al.*, 2013 ▸; Sorzano *et al.*, 2015 ▸), which uses electron atomic scattering factors. Two data sets were prepared with the aim of studying different resolution ranges: (i) 3D density maps simulated with a sampling rate of 1.0 Å per voxel (data set 1) and (ii) simulated maps with a sampling rate of 0.5 Å per voxel (data set 2). A description in more concrete terms is given below.

*Data set 1*. For each 3D map, a filter bank was created so that it was low-pass filtered to frequencies of between 2.5 and 13.0 Å (every 0.1 Å) with a raised cosine of 0.02 (in normalized units) using the *xmipp_transform_filter* function.
*Data set 2*. As data set 1, but in this case maps were low-pass filtered to frequencies of between 1.5 and 6.0 Å (every 0.1 Å) with a raised cosine of 0.02 (normalized units). The reason for creating this second data set was to analyze maps at very high resolution.

In order to locally study these maps, each filtered map was divided into boxes of 13 × 13 × 13 voxels, which correspond to edge lengths of 13 and 6.5 Å for data set 1 and data set 2, respectively. The density values of each box were normalized to have a unit norm and guarantee uniform intensity ranges of all filtered maps. Millions of simulated map boxes and their corresponding resolution labels were then used to train the neural network.

### 2.2. Training of the *DeepRes* network   

*DeepRes* trains a convolutional neural network (CNN) for the automatic estimation of local quality. The CNN was implemented using the Keras 2.0.2 (http://keras.io) Python deep-learning library with the TensorFlow (Abadi *et al.*, 2016 ▸) backend. Our CNN contained a convolutional layer that applied 32 13 × 13 × 13 filters (many other architectures were tested with similar results, but this was the simplest one tested, which was why we selected it), followed by a dense layer with 512 neurons and the output dense layer with just one neuron reporting the local quality label (note that we are addressing *DeepRes* as a regression analysis, rather than as a classification). We used the rectified linear unit (RELU) as an activation function. The padding ‘same’ was used following each layer to preserve the map dimensions. Dropout with a probability of 0.25 was applied to the convolutional layer output to regularize the network and avoid overfitting. To optimize the network parameters, we used the Adam optimizer (Kingma & Ba, 2014 ▸), which is an improved version of stochastic gradient descent. The optimizer determines how the gradient of the loss function is used to update the network parameters. In *DeepRes*, the loss function that is minimized is ‘mean_squared_error’.

### 2.3. *DeepRes* input and output   

The algorithm requires as input a 3D cryoEM density map and a mask enclosing the macromolecule. These input maps are rescaled to a pixel size of 1.0 or 0.5, depending on the CNN that is going to be used. The map is then sampled in a sliding window of 13 × 13 × 13 voxels. Finally, the resolution estimate is assigned to the voxel located at the center of the cube.

### 2.4. Code availability   

*DeepRes* is publicly available from *Xmipp* (de la Rosa-Trevín *et al.*, 2013 ▸; http://xmipp.cnb.csic.es) in the development branches https://github.com/I2PC/xmipp/ and https://github.com/I2PC/scipion-em-xmipp/ (these branches will eventually become the next release of *Xmipp*), and is integrated into the image-processing framework *Scipion* (de la Rosa-Trevín *et al.*, 2016 ▸; http://scipion.cnb.csic.es). A tutorial on how to use *DeepRes* can be found at https://github.com/I2PC/scipion-em-xmipp/wiki/DeepRes-local-resolution.

## 3. Results   

Different visualization options have been implemented into *Scipion* (de la Rosa-Trevín *et al.*, 2013 ▸) to analyze the results [for example, the local resolution map, a resolution histogram, colored slices of the resolution map and the display of the original map in *UCSF Chimera* (Pettersen *et al.*, 2004 ▸) colored according to the obtained resolution values]. The validation of *DeepRes* was initially carried out through simulated maps for different scenarios. This exercise allows us to evaluate the method using maps for which the local resolution values are known *a priori*. The method was subsequently applied to different experimental maps and the results were compared using current methods of estimating local resolution (*ResMap*, *MonoRes* and *BlocRes*). For all methods, the same mask and default parameters were used. In *ResMap*, manual pre-whitening was performed to the best of our knowledge.

### 3.1. Tests with simulated data   

In a first step to test the performance of *DeepRes*, the method was applied to two simulated maps that were not employed for training and for which local resolutions were known. For the first case we used the atomic model of the 39 kDa human cartilage glycoprotein tetramer (HCGP39; PDB entry 1hjv; Houston *et al.*, 2003 ▸). For this structure, we generated a map with a sampling rate of 1.0 Å per pixel using *xmipp_volume_from_pdb* (Sorzano *et al.*, 2015 ▸). Each monomer in the structure was selected and low-pass filtered at different frequencies of 3, 5, 7 and 9 Å with a raised cosine of 0.02 (in normalized units). After this, Gaussian noise with zero mean and a standard deviation (SD) of 0.08 was added.

**Figure 1:**
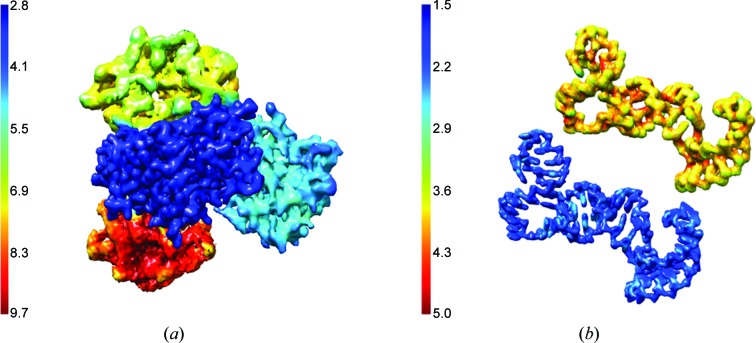
*DeepRes* results for simulated maps. (*a*) *DeepRes* resolution map from the simulated map of the 39 kDa human cartilage glycoprotein tetramer filtered at 3, 5, 7 and 9 Å (PDB entry 1hjv). (*b*) *DeepRes* resolutions maps from two simulated maps of φ29 pRNA (PDB entry 3r4f) filtered at 2 and 4 Å.

The results of the *DeepRes* application in this test are shown in Fig. 1 ▸(*a*). Our method is capable of capturing the different characteristics within the map and provides a resolution map with values that match the cutoff frequencies. In particular, the median values obtained with *DeepRes* were 3.2, 5.0, 7.1 and 8.9 Å, with SDs of 0.2, 0.3, 0.5 and 0.5 Å, respectively.

The second simulated test considered the crystal structure of the φ29 pRNA prohead-binding domain (PDB entry 3r4f; Ding *et al.*, 2011 ▸). This case also allows us to check whether the method works well for nucleotides. As before, the atomic model was converted into a density volume with a sampling rate of 0.5 Å per pixel. Two low-pass-filtered maps were then generated at frequencies of 2 and 4 Å with a raised cosine of 0.02. Noise was added as in the previous case.

Fig. 1 ▸(*b*) shows that *DeepRes* produces quality measures close to the expected resolution values. For the map filtered at 2 Å the median resolution value estimated was 2.3 Å with a standard deviation (SD) of 0.3 Å [Fig. 1 ▸(*b*), blue], while for the map filtered at 4 Å the median resolution was 4.0 Å with an SD of 0.3 Å [Fig. 1 ▸(*b*), yellow].

The results obtained for the simulated data confirm that *DeepRes* estimates local resolutions that are very close to the expected theoretical values and validate the applicability of our method for macromolecules that contain both amino acids and nucleotides.

Additionally, the second simulated map (φ29 pRNA) was also used to investigate the basis of our method. Both *MonoRes* and *ResMap* are based on a comparison between the energy of the signal and the energy of the noise. To study whether *DeepRes* was only taking into account the frequential energy of the map, without any further connection to the underlying macromolecular structure, a test based on Fourier phase randomization was carried out. Thus, in the map filtered at 2 Å, the phases were randomized beyond 4 Å. The results of *DeepRes* for the original map and the phase-randomized map are shown in Supplementary Fig. S2. Clearly, when randomization is applied, *DeepRes* shifts the resolution values to 4 Å. These results show that *DeepRes* not only takes into account the energy but also detects the difference in texture in the analyzed maps.

### 3.2. Results on experimental maps   

**Figure 2:**
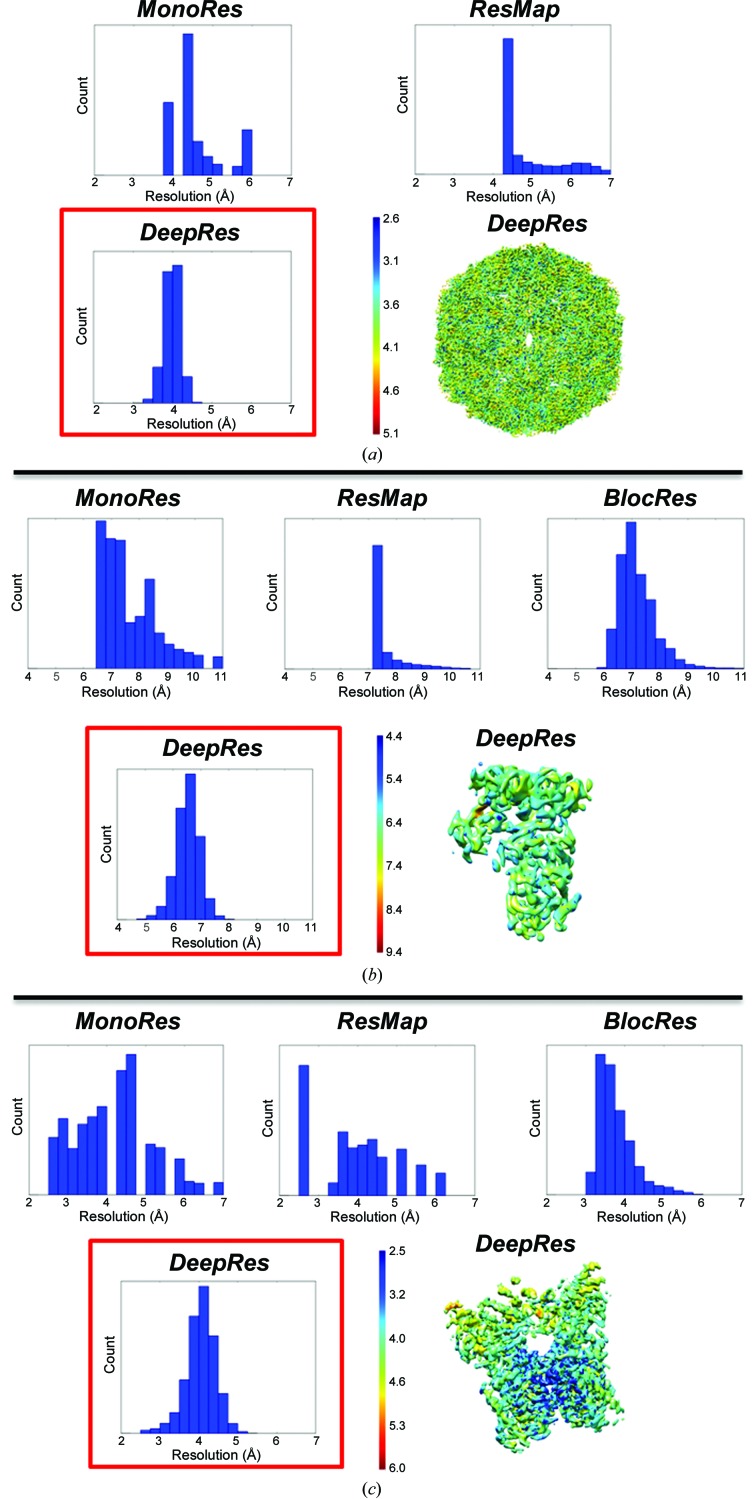
Local resolution results for experimental maps. The resolution histograms obtained by the different methods (*MonoRes*, *ResMap*, *BlocRes* and *DeepRes*) and the resolution map determined with *DeepRes* are shown for (*a*) enterovirus D68 (EMDB entry EMD-9631), (*b*) the PolIIIα–clamp–exonuclease–θ DNA complex (EMDB entry EMD-4141) and (*c*) capsaicin receptor TRPV1 (EMDB entry EMD-5778).

Once the effectiveness of our method for estimating local resolution with simulated maps had been proven, we applied *DeepRes* to five cryoEM maps and the results were compared using *MonoRes*, *ResMap* and *BlocRes* (Fig. 2 ▸ and Supplementary Fig. S3). The five analyzed maps (enterovirus D68, the PolIIIα–clamp–exonuclease–θ DNA complex, capsaicin receptor TRPV1, HSP104_DWB_ and CMG helicase) were obtained from the EMDB (EMDB entries EMD-9631, EMD-4141, EMD-5778, EMD-0376 and EMD-3320, respectively) and correspond to maps that had been subjected to global *B*-factor (Rosenthal & Henderson, 2003 ▸) post-processing in *RELION* (Scheres, 2012 ▸).

#### 3.2.1. Enterovirus D68   

The first experimental case analyzed the map of enterovirus D68 (EMDB entry EMD-9631; Zheng *et al.*, 2019 ▸) with a resolution of 4.0 Å as reported by the gold-standard FSC of 0.143. The resolution histograms obtained using *DeepRes*, *MonoRes* and *ResMap* are shown in Fig. 2 ▸(*a*). *BlocRes* was not applied in this test because the half maps were not deposited in the EMDB. *DeepRes* [red square in Fig. 2 ▸(*a*)] estimated the resolution values in a narrow range from 3.4 to 4.6 Å, with the median at 4.0 Å and an SD of 0.2 Å. This median resolution is in total agreement with the reported resolution of 4.0 Å (Table 1 ▸). The resolution map obtained with *DeepRes* is represented using *UCSF Chimera* in Fig. 2 ▸(*a*). The distribution with *MonoRes* ranges from 4.0 to 6.0 Å, with the median at 4.5 Å and an SD of 0.6 Å. Using *ResMap* a peak of resolution was obtained at 4.4 Å and values extended up to 7.0 Å, presenting a median resolution of 4.4 Å and an SD of 0.8 Å.

#### 3.2.2. PolIIIα–clamp–exonuclease–θ DNA   

The second case corresponds to the PolIIIα–clamp–exonuclease–θ DNA complex (EMDB entry EMD-4141; Fernandez-Leiro *et al.*, 2017 ▸). The reported resolution for this map was 6.7 Å (gold-standard FSC of 0.143). The results obtained using the different methods are shown in Fig. 2 ▸(*b*). With *MonoRes*, the resolution range obtained ranges from 6.6 to 11 Å, with a median of 7.5 Å and an SD of 1.1 Å, although the highest peak in the distribution is between 6.6 and 7.4 Å. *ResMap* estimated a resolution peak at 7.4 Å with an SD of 0.6 Å, while the histogram of resolutions calculated with *BlocRes* is centered at 7.0 Å with an SD of 0.7 Å. The histogram representing the resolution values obtained using *DeepRes* is centered at 6.7 Å, with a median of 6.5 Å and an SD of 0.4 Å. As in the previous case, the median resolution of *DeepRes* is close to the reported FSC resolution (Table 1 ▸).

#### 3.2.3. Capsaicin receptor TRPV1   

The third experimental map used corresponds to the well known membrane protein TRPV1 (EMDB entry EMD-5778; Liao *et al.*, 2013 ▸). The reported resolution for this density map was 3.4 Å at the gold-standard FSC of 0.143. This is an interesting case of a membrane protein with a wide range of local resolutions. We took special care to mask out the membrane and work only with the macromolecular complex, which is certainly a general procedure, but in our case it was especially important since *DeepRes* was only trained with proteins. The results and the comparison with current methods are shown in Fig. 2 ▸(*c*). *DeepRes* reported a median resolution of 4.1 Å with an SD of 0.4 Å, while these values were 4.3 ± 1.1, 4.0 ± 1.0 and 3.7 ± 0.5 Å for *MonoRes*, *ResMap* and *BlocRes*, respectively. As expected, *DeepRes* detected a high-resolution area corresponding to the center of the transmembrane region (<3.8 Å) and a lower resolution area on the ankyrin motif (>4.7 Å).

#### 3.2.4. Low-resolution maps: HSP104_DWB_ and CMG helicase   

Two cases were used to validate the use of *DeepRes* with low-resolution maps. The first corresponds to the HSP104_DWB_ map (EMDB entry EMD-0376; Lee *et al.*, 2019 ▸) with a reported resolution of 9.3 Å at the gold-standard FSC of 0.143 [Supplementary Fig. S3(*a*)]. The histogram obtained with *MonoRes* shows resolutions above 8 Å, with a maximum at 9.6 Å and a median of 11.7 Å (Table 1 ▸), while the resolutions obtained with *ResMap* are above 10 Å with a median of 10.2 Å. The highest number of values obtained with *DeepRes* are between 8 and 11 Å resolution, with a median resolution of 9.3 Å and an SD of 1.2 Å (Table 1 ▸), which matches the FSC resolution reported for the map.

The second case corresponds to the CMG helicase map (EMDB entry EMD-3320; Abid Ali *et al.*, 2016 ▸) with a reported resolution of 10.2 Å at the gold-standard FSC of 0.143 [Supplementary Fig. S3(*b*)]. In this case, the current methods could not be applied because the deposited map was masked and free of noise. The median resolution obtained by *DeepRes* is 10.1 Å with an SD of 1.7 Å (Table 1 ▸), which is in agreement with the resolution reported by the authors.

### 3.3. Local resolutions from unsharpened and sharpened maps   

One of the main limitations of the current methods is that they are not able to cope with situations such as differentiating between unsharpened and sharpened maps. Indeed, both the FSC as well as the local resolution determined by the monogenic signal (Vilas *et al.*, 2018 ▸) and the local sinusoids versus noise (Kucukelbir *et al.*, 2014 ▸) are insensitive to isotropic, non­vanishing filters (Unser *et al.*, 2005 ▸; Sorzano *et al.*, 2017 ▸; see Supplementary Fig. S1). In particular, when a global *B*-factor-based sharpening is applied, all spectral components at a given radial frequency are modified proportionally (Unser *et al.*, 2005 ▸) and therefore the statistically detected frequency above the noise level will remain invariant.

**Figure 3:**
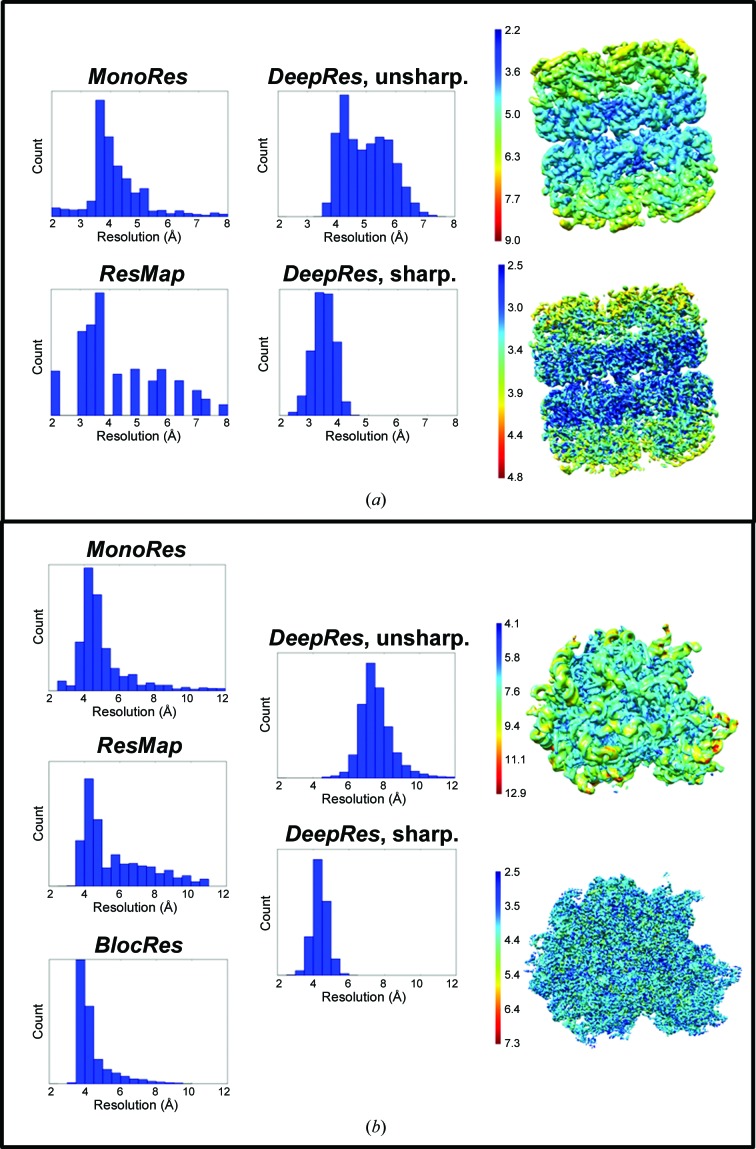
Changes in local resolution between unsharpened maps and sharpened maps detected with *DeepRes*. Histograms determined with *MonoRes*, *ResMap* and *BlocRes* for unsharpened maps (left), the histograms determined for unsharpened and sharpened maps (center) with *DeepRes* and the resolution maps for unsharpened and sharpened maps (right) of (*a*) *E. coli* GroEL (EMDB entry EMD-3407) and (*b*) the rabbit 80S ribosome (EMDB entry EMD-9235). The sharpened maps were obtained with *LocalDeblur* and *RELION* post-processing for GroEL and ribosome, respectively. Note that the color scales of the resolution maps of the different specimens have been modified for better visualization.

**Figure 4:**
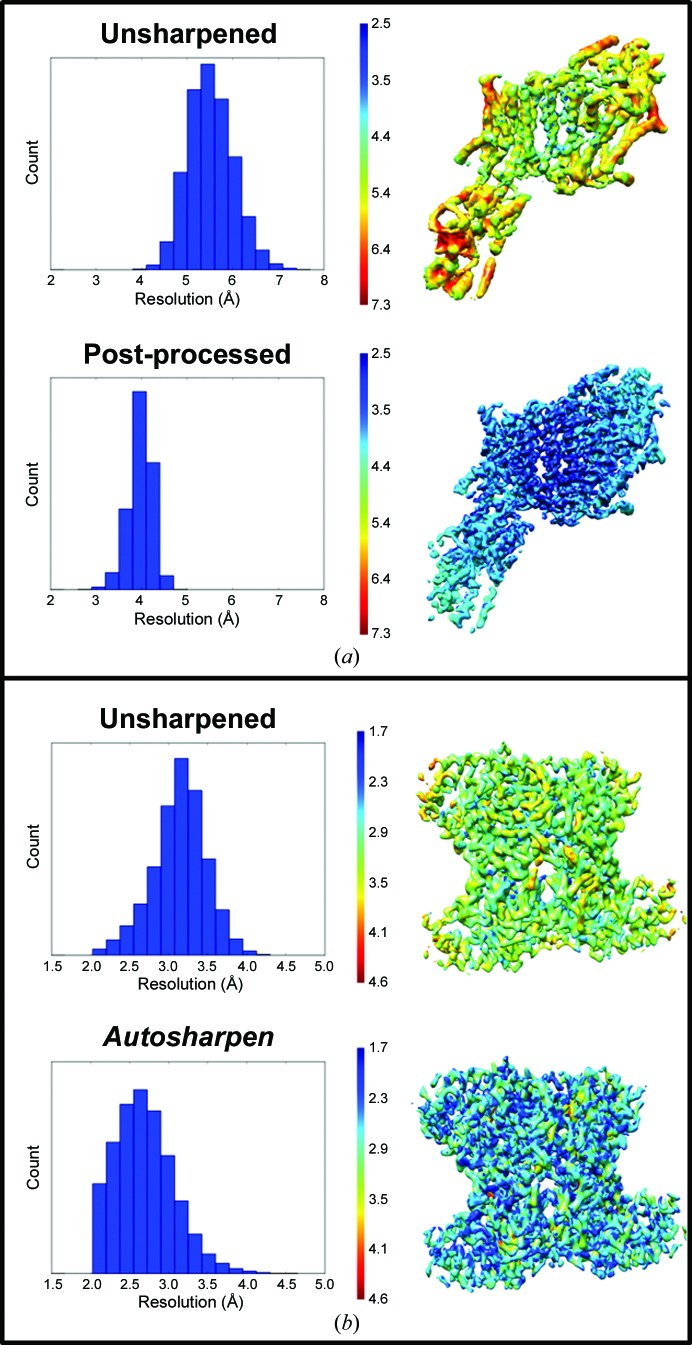
*DeepRes* results from unsharpened and sharpened maps. Histograms (left) and resolution maps (right) for sharpened and unsharpened maps of (*a*) the KdpFABC complex (EMDB entry EMD-0258) and (*b*) rabbit muscle aldolase (EMDB entry EMD-7550). The sharpened maps were obtained with *RELION* post-processing and *Autosharpen* for KdpFABC and muscle aldolase, respectively.

However, resolution changes after applying *B*-factor sharpening can be detected using *DeepRes*. Other maps that will benefit from *DeepRes* are those obtained with methods that modify the signal or minimize the noise level [for example, *LocalDeblur* (Ramírez-Aportela *et al.*, 2019 ▸) or *Xmipp Highres* (Sorzano *et al.*, 2018 ▸)]. Resolution changes determined with *DeepRes* before and after map sharpening are exemplified in Fig. 3 ▸ and Supplementary Fig. S4 with three different experimental cases, *Escherichia coli* GroEL, rabbit 80S ribosome and *Plasmodium* 80S ribosome maps, and in Fig. 4 ▸ with both the KdpFABC complex and rabbit muscle aldolase.

#### 3.3.1. *E. coli* GroEL   

The first case is based on the *E. coli* GroEL map in the apo form (EMDB entry EMD-3407; Joseph *et al.*, 2016 ▸) reconstructed at 3.3 Å resolution (gold-standard FSC of 0.143); in this case the unsharpened map was deposited and will be the map used in our first set of analyses. For the unsharpened map, *MonoRes* and *ResMap* estimated resolution ranges from 2.25 to 8.0 and 2.3 to 8.0 Å, with medians of 4.0 and 3.8 Å, respectively [Fig. 3 ▸(*a*) and Table 1 ▸]. However, the results with our method are markedly different. *DeepRes* detected resolutions ranging from 3.3 to 7.0 Å, with a median resolution of 5.0 Å. That is, when applied to the unsharpened map *DeepRes* reported resolutions that were lower than with any other methods.

We then proceed to sharpen the map using *LocalDeblur* (Ramírez-Aportela *et al.*, 2019 ▸) and to determine the local resolution of GroEL using *DeepRes* [Fig. 3 ▸(*a*)]. Note that, as previously indicated, the use of algorithms such as *MonoRes* or *ResMap* on these modified maps is not mathematically justified (see the supporting information). Interestingly, *DeepRes* shows a clear increase in resolution with respect to the unsharpened map, estimating the resolution to be in the range 2.5–4.5 Å, with a median resolution of 3.5 Å. In general, *DeepRes* reports an average resolution gain for each domain of greater than 1.0 Å with respect to the unsharpened map. Note that the results obtained for the sharpened map using *DeepRes* are of the same order as the results obtained by the other methods for the unsharpened map (this observation will be further commented on in Section 4 and in the supporting information). Consequently, the results of *DeepRes* are very intuitive, which is not the case for the other approaches.

#### 3.3.2. Rabbit 80S ribosome and *Plasmodium* 80S ribosome   

The second case corresponds to the rabbit 80S ribosome map (EMDB entry EMD-9235; Brown *et al.*, 2018 ▸), with a reported overall resolution of 3.8 Å (gold-standard FSC of 0.143). Note that in this case the original deposition contained the two half maps, so that *BlocRes* could also be applied. The results for this unsharpened density map also showed clear discrepancies between *DeepRes* and the current methods [Fig. 3 ▸(*b*)]. While *MonoRes*, *ResMap* and *BlocRes* estimated median resolutions of 4.5, 4.9 and 4.0 Å, respectively, *DeepRes* estimated a median resolution of 7.4 Å (Table 1 ▸).

When we determined the local resolution with *DeepRes* for the deposited map (Brown *et al.*, 2018 ▸) post-processed with *RELION*, an increase in the resolution with respect to the unsharpened map was detected [Fig. 3 ▸(*b*)]. The histogram shows a resolution range from 3.1 to 6.0 Å, with a median of 4.4 Å and an SD of 0.4 Å. As for the GroEL maps, *DeepRes* detected a noticeable change in resolution after post-processing, so that the *DeepRes*-estimated local resolution values of the sharpened map are in the range of the resolutions estimated by the other methods.

A similar behavior is shown for the *Plasmodium* 80S ribosome map (EMDB entry EMD-2660; Wong *et al.*, 2014; Supplementary Fig. S4). The unsharpened map presents a resolution range lower than those determined by the current methods, with a median resolution of 6.3 Å and an SD of 1.3 Å (Table 1 ▸). Moreover, the *DeepRes* histogram for the sharpened map with *Autosharpen* (Terwilliger *et al.*, 2018 ▸) presents a resolution range similar to the other methods, with a median resolution of 3.8 Å and an SD of 0.9 Å. Both the sharpened and the unsharpened maps have a low resolution for the head region of the small 40S subunit owing to its inherent flexibility, as reported by the authors (Wong *et al.*, 2014 ▸).

These changes are related to the capacity of *DeepRes* to detect increments in local resolution after sharpening, which the other approaches cannot detect. We will particularize the conceptually simple case of a global *B*-factor correction. Indeed, FSC, *ResMap* and *MonoRes* are intrinsically insensitive to quasi-flattening the spectrum of the map, since it affects the noise and signal equally. These methods are designed to extract correlations or SNRs irrespective of this enhancement; in a way, they already provide the best value that any simultaneous enhancement of signal and noise per frequency can obtain (see the detailed analysis presented in Supplementary Fig. S1 and its respective section). On the contrary, *DeepRes* is sensitive to these changes, so that when sharpening is applied the resolution improves and it then becomes very similar to the results of all other methods.

#### 3.3.3. Other maps: the KdpFABC complex and rabbit muscle aldolase   

Two other cases were taken into account to evaluate the resolution when sharpening is applied (Fig. 4 ▸). One of the cases tested was the KdpFABC complex (EMDB entry EMD-0258; Stock *et al.*, 2018 ▸). The original publication reported a resolution of 4.0 Å (gold-standard FSC of 0.143). Our *DeepRes* resolution estimates are between 4.0 and 7.0 Å for the unsharpened map and between 3.0 and 5.0 Å for the post-processed map [Fig. 4 ▸(*a*)]. The median resolution varied from 5.5 to 4.0 Å with post-processing, with the latter value being similar to that reported by the FSC (Table 1 ▸). In both maps, a better resolution zone (belonging to the KdpA domain) is observed with resolutions between 4.4 and 5.5 Å for the original map and between 3.0 and 4.0 Å for the sharpened map, and a lower resolution zone (belonging to KdpC and the N, P and A cytoplasmatic domains of KdpB) with resolutions between 6.2 and 7.0 Å for the original map and 4.3 and 5.0 Å for the sharpened map.

Finally, we analyzed a rabbit muscle aldolase reconstruction (EMDB entry EMD-7550; Kim *et al.*, 2018 ▸). The original publication estimated a resolution of 2.4 Å (gold-standard FSC of 0.143). In this case, a comparison of our method was made with the unsharpened map and the map after having applied *Autosharpen* [Fig. 4 ▸(*b*)]. Both maps show resolutions that vary between 2.0 and 4.0 Å, but the median changed from 3.2 to 2.6 Å on the application of *Autosharpen*.

### 3.4. Oversharpening detection   

**Figure 5:**
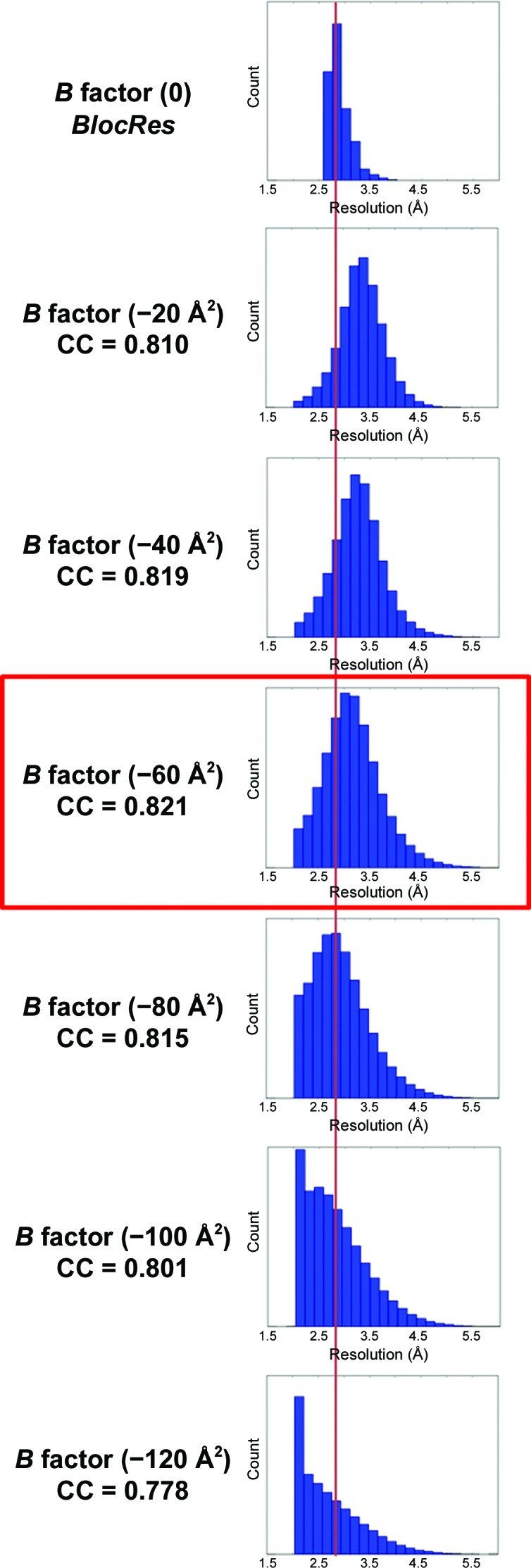
Oversharpening detection. Comparison of *BlocRes* applied to the unsharpened map (top of the figure) and *DeepRes* applied to each *B*-­factor sharpened map. The correlation (CC) of each sharpened map and the generated map from the deposited atomic model (PDB entry 6bdf) was calculated using *PHENIX*. The red line corresponds to the 20% percentile of the local resolution distribution of *BlocRes*.

The current methods (all of which are based on different ways of estimating the SNR) report the higher resolution of the map without detecting the degree of blurring, which results in a downweighting of the high-frequency components. Indeed, the choice of the applied *B* factor modifies the appearance of the map, but this change is not detected by the current methods because it is a radially symmetric operation. On the other hand, if a map is oversharpened then the resolution measures based on the SNR are not affected either. Unlike these methods, *DeepRes* is capable of detecting differences in resolution between the unsharpened and sharpened maps, which allow the sharpening quality to be evaluated. In the case of oversharpening, *DeepRes* would report an overestimation of the resolution. Consequently, a good strategy to detect and avoid oversharpening the maps is the combination of both kinds of methods, as exemplified in Fig. 5 ▸ and Supplementary Figs. S5 and S6. If the resolution reported by *DeepRes* is higher than the resolution reported by an SNR-based method then this is an indicator of oversharpening.

One of the monomers in the structure of the 39 kDa human cartilage glycoprotein tetramer (HCGP39; PDB entry 1hjv, chain *A*) was used to generate a map with a sampling rate of 1.0 Å per pixel. This map was low-pass filtered at a frequency of 3.5 Å with a raised cosine of 0.02. Gaussian noise with zero mean and an SD of 0.08 was added. The map was then sharpened using negative *B* factors of −60 and −100 Å^2^ and the resolution of the maps was determined using *MonoRes* and *DeepRes* (Supplementary Fig. S5). With *MonoRes* the resolution remained practically invariant, while with *DeepRes* the detected resolution increased and exceeded the limit determined by *MonoRes* (based on the SNR). This example demonstrates that the combination of methods is useful for detecting the oversharpening of density maps.

This strategy to detect oversharpening was applied to the 20S proteasome map (EMDB entry EMD-6287; Campbell *et al.*, 2015 ▸; Fig. 5 ▸ and Supplementary Fig. S6). Several *B* factors were applied to the unsharpened map, and the correlations of each map and the generated map from the deposited atomic model (PDB entry 6bdf) were calculated using *PHENIX* (Afonine *et al.*, 2018 ▸). Among the filtered maps, the maximum correlation was obtained for the sharpened map with a *B* factor of −60 Å^2^. The local resolution of the sharpened maps was then determined with *DeepRes* and compared with the local resolution obtained by *BlocRes* from the two halves of the unsharpened map. Without the atomic model, it would be impossible in a real experiment to know which is a global *B* factor that does not cause oversharpening. Using *DeepRes*, we have heuristically observed that a good *B* factor is one such that the 20% percentile of the local resolution distribution of *BlocRes* or *MonoRes* (unsharpened) and *DeepRes* (sharpened) coincide, as illustrated in Fig. 5 ▸ for *BlocRes* and in Supplementary Figs. S6 for *MonoRes*. In the example shown, the heuristic non-oversharpening *B* factor is −60 Å^2^ according to *BlocRes* and *MonoRes*.

## 4. Discussion   

In this work, we have introduced a new approach aimed at estimating the local quality of a map using principles totally different from any other method previously used in the field, in which we have used deep learning. Our motivation to propose ‘still another’ local quality measure (another ‘local resolution’) is very simple and stems from the fact that most resolution-estimation methods currently used are intrinsically insensitive to isotropic, nonvanishing Fourier filters (by non­vanishing we mean that they do not set to zero-frequency components). Interestingly, this characteristic has been very little treated in the cryoEM literature [with exceptions, such as Unser *et al.* (2005 ▸) and Sorzano *et al.* (2017 ▸)], but effectively it means that transformations that change the spectrum of a macromolecule (for instance, by enhancing the amplitude of its Fourier components) are not detected by current methods or, in other words, the resolution values that they report will be the same before and after this enhancement. This result is totally counterintuitive, but it is rigorously demonstrated in depth in the supporting information, together with an illustrative example (Supplementary Fig. S1). Note that the comments above refer to the use of filters only, not to the combination of filters with other operations such as, for example, changing the mask (for example, making a mask tighter). We wanted to develop an approach that naturally renders lower quality values when maps have not been ‘enhanced’ and higher ones after ‘enhancement’, without any change of parameters or masks or any other operation.

We have used deep-learning technology to ‘teach’ a neural network the characteristics of filtered maps at different resolutions. Initially, our approach was tested with simulated maps in which the resolution values were known *a priori*. In these tests different scenarios were studied: maps with different resolutions and different types of macromolecules (amino acids and nucleotides). The results of *DeepRes* were excellent for the cases tested, assigning resolutions very similar to the expected values and validating the use of our method to estimate the local resolution in density maps.

However, the resolution values obtained with *DeepRes* for known experimental unsharpened maps were much more conservative than those estimated using the current methods. Still, when sharpening was applied to these maps, the *DeepRes* resolutions were in accordance with the current methods. The resolution medians obtained by *DeepRes* for the tested sharpened maps were also close to the resolutions reported by FSC.

As previously indicated, the current methods are insensitive to the application of a *B* factor, which means that different modifications of the map by the application of different *B*-factor values will not be reflected by a change in resolution. In this sense, our method offers a solution. With *DeepRes*, the changes that occur with an applied *B* factor are indeed detected. The results obtained indicate that the resolutions estimated by the current methods correspond to the resolutions that the map would have after restoration. However, our method reports the resolution as the similarity to biological macromolecules filtered at a given resolution, which is closer to the user observation that sharpening facilitates the atomic modeling of the macromolecule. We have also shown that comparison of the current methods (based on SNR) and *DeepRes* allows detection of the oversharpening of cryoEM maps.

Another advantage of *DeepRes* is that it does not depend on map noise estimations. Therefore, *DeepRes* can be used for maps with masks applied, with a low noise level owing to the processing technique or to locally enhanced signal using algorithms such as *LocScale* and *LocalDeblur*.

## Supplementary Material

Insensitivity of resolution methods to isotropic filters, limitation of methods based on signal-to-noise ratio for maps affected by noise suppression and Supplementary Figures. DOI: 10.1107/S2052252519011692/fq5008sup1.pdf

## Figures and Tables

**Table 1:** Summary of median resolution for the experimental cases

EMDB code	FSC (Å)	*MonoRes *median (Å)	*ResMap* median (Å)	*BlocRes* median (Å)	*DeepRes* sharpened median (Å)	*DeepRes* unsharpened median (Å)
EMD-9631	4.0	4.5 ± 0.6	4.4 ± 0.8	—	4.0 ± 0.2	—
EMD-4141	6.7	7.5 ± 1.1	7.4 ± 0.6	7.0 ± 0.7	6.5 ± 0.4	—
EMD-5778	3.4	4.3 ± 1.1	4.0 ± 1.0	3.7 ± 0.5	4.1 ± 0.14	—
EMD-0376	9.3	11.7 ± 2.3	10.2 ± 0.8	—	9.3 ± 1.2	—
EMD-3320	10.2	—	—	—	10.1 ± 1.7	—
EMD-3407	3.3	4.0 ± 1.1	3.8 ± 1.5	—	3.5 ± 0.4	5.0 ± 0.8
EMD-9235	3.8	4.5 ± 1.8	4.9 ± 1.8	4.0 ± 1.0	4.4 ± 0.4	7.4 ± 1.0
EMD-2660	3.2	4.0 ± 2.7	4.6 ± 1.7	3.6 ± 1.1	3.8 ± 0.9	6.3 ± 1.3
EMD-0258	4.0	—	—	—	4.0 ± 0.3	5.5 ± 0.5
EMD-7550	2.4	—	—	—	2.6 ± 0.4	3.2 ± 0.3
