# Supplementary material for: DeepRes: a new deep-learning- and aspect-based local resolution method for electron-microscopy maps
Source: IUCrJ. 2019 Sep 18;6(Pt 6):1054–63. doi: 10.1107/S2052252519011692 (PMC6830216; doi:10.1107/S2052252519011692)
Supplement: Supplementary file 1 [file m-06-01054-sup1.pdf]

IUCrJ

Volume 6 (2019)

Supporting information for article:

DeepRes: a new deep learning- and aspect-based local resolution method for electron-microscopy maps

Erney Ramírez-Aportela, Javier Mota, Pablo Conesa, Jose Maria Carazo and Carlos Oscar S. Sorzano

S1. Insensitivity of resolution methods to isotropic filters (B-factor correction)

S1.1. Fourier Shell Correlation (FSC)

The FSC is currently the most widely used algorithm to determine the resolution of CryoEM maps (Saxton & Baumeister, 1982, Saxton, 1978, Harauz & van Heel, 1986). To measure the FSC curve, two independently determined 3D volumes are required. This algorithm defines the resolution as the maximum frequency at which the correlation between both is above a given threshold. Based on this principle, but with a moving window of a previously fixed size, BlocRes (Cardone *et al.*, 2013) was proposed to determine the local resolution of cryoEM maps. However, as described by Unser and colleague s(Unser *et al.*, 2005) and Sorzano and colleagues (Sorzano *et al.*, 2017); the FSC is invariant to isotropic filters, therefore being invariant to a transformation of the type B-factor correction (Rosenthal & Henderson, 2003). Despite this, it is known that CryoEM maps significantly benefit of a post-processing step (sharpening) that allows increase signal at medium/high resolution, and qualitatively improve map quality. One of the most widely used methods for sharpening is just the correction for a negative B-factor, and most of the maps deposited in EMDB are the result of this processing step. Although the quality of the map is significantly improved with postprocessing, the FSC and all its derived measured are insensitive to the change produced (unless other changes are introduced at the same time such as, for instance, at the masking level). The insensitivity of the FSC to an isotropic filter (as B-factor correction) is shown below.

Consider two independent reconstructed density maps f_1 and f_2, being F_1 and F_2 the Fourier transform of f_1 and f_2 respectively, the FSC is normally defined as

$$FSC(R, \Delta R) = \frac{\sum_{\boldsymbol{R}\in\mathfrak{S}(\mathrm{R},\Delta R)} F_1(\boldsymbol{R})\, F_2^*(\boldsymbol{R})}{\sqrt{\left(\sum_{\boldsymbol{R}\in\mathfrak{S}(\mathrm{R},\Delta R)} |F_1(\boldsymbol{R})|^2\right)\left(\sum_{\boldsymbol{R}\in\mathfrak{S}(\mathrm{R},\Delta R)} |F_2(\boldsymbol{R})|^2\right)}}$$

where **R** is the three-dimensional frequency vector, R its module and $\mathfrak{S}(\mathrm{R}_0, \Delta\mathrm{R})$the shell of those frequencies such that $R_0 \le \|\boldsymbol{R}\| < R_0 + \Delta R$.

Consider H(**R**) as an isotropic filter that acts on the volumes f_1 and f_2 (**H**F_1 and **H**F_2), then the FSC is defined as

$$FSC_H(R, \Delta R) = \frac{\sum_{\boldsymbol{R}\in\mathfrak{S}(\mathrm{R},\Delta R)} H(\boldsymbol{R})F_1(\boldsymbol{R})H^*(\boldsymbol{R})F_2^*(\boldsymbol{R})}{\sqrt{\left(\sum_{\boldsymbol{R}\in\mathfrak{S}(\mathrm{R},\Delta R)} |H(\boldsymbol{R})F_1(\boldsymbol{R})|^2\right)\left(\sum_{\boldsymbol{R}\in\mathfrak{S}(\mathrm{R},\Delta R)} |H(\boldsymbol{R})F_2(\boldsymbol{R})|^2\right)}}$$

$$= \frac{|H(R)|^2 \sum_{\boldsymbol{R}\in\mathfrak{S}(\mathrm{R},\Delta R)} F_1(\boldsymbol{R})\, F_2^*(\boldsymbol{R})}{|H(R)|^2 \sqrt{\left(\sum_{\boldsymbol{R}\in\mathfrak{S}(\mathrm{R},\Delta R)} |F_1(\boldsymbol{R})|^2\right)\left(\sum_{\boldsymbol{R}\in\mathfrak{S}(\mathrm{R},\Delta R)} |F_2(\boldsymbol{R})|^2\right)}} = FSC(R, \Delta R)$$

Note that as H(**R**) is an isotropic filter, then $\mathrm{H}(\mathbf{R}) = \mathrm{H}(\mathrm{R}) \in \mathbb{R}$ and $\mathrm{FSC}(\mathrm{R}, \Delta\mathrm{R}) = \mathrm{FSC_H}(\mathrm{R}, \Delta\mathrm{R})$, being the FSC curve insensitive to the filter application (in this case B-factor correction).

Supplementary Figure 1A shows the aforementioned. The crystal structure of ϕ29 pRNA prohead-binding domain (PDB: ID-3R4F) (Ding *et al.*, 2011) was converted into a density volume with sampling rate of 0.5 Å/pixel, low-pass filtered at 4 Å. Then, two maps were created by adding Gaussian noise with zero mean and 0.08 SD. These two maps were considered to calculate the FSC curve (red profile in Supplementary Figure S1). Subsequently, a B-factor correction was applied to these two maps, applying a B-factor of -60, and the new FSC (gray profile in Supplementary Figure 1) was determined. As shown in the figure, the FSC curve does not change with the correction.

S1.2. Methods based on signal-to-noise ratio

Other local methods define the resolution as the maximum detectable frequency above the noise power. In this principle, although using different algorithms, MonoRes (Vilas *et al.*, 2018) and Resmap (Kucukelbir *et al.*, 2014) are based. These methods also have limitations to detect the quality changes produced by applying a B-factor correction. When an isotropic filter (as B-factor correction) is applied, all spectral components at a given radial frequency are modified proportionally and therefore, the signal-to-noise ratio does not change.

In Supplementary Figure 1B we exemplify this fact for the particular case of MonoRes. In this test, the simulated map (low-pass filtered at 4 Å) from the structure of ϕ29 pRNA prohead-binding domain previously introduced in the main section was used. As shown in the figure, the resolution map estimated by MonoRes is similar to the estimated resolution map when a correction with a B-factor of -60 is used.

S2. Limitation of methods based on the signal-to-noise ratio for maps affected by noise suppression

The need to determine the variance of the noise to estimate the local resolution is the main limitation of methods like MonoRes or ResMap. Not only because differentiating between noise and signal can be a challenge, but also because in some cases the processing methods apply techniques of noise suppression and signal enhancement, as in the case of algorithms such as HighRes (Sorzano *et al.*, 2018) or LocalDeblur (Ramirez-Aportela *et al.*, 2018). In these cases the application of methods based on SNR could cause an overestimation of resolution.

Supplementary Figure 1C shows the results of applying DeepRes, MonoRes and Resmap to the LocalDeblur map of *E. coli* GroEL (EMD-3407) (Joseph *et al.*, 2016). The resolutions estimated by MonoRes and ResMap are close to the Nyquist limit, while with DeepRes a distribution with median of 3.5 Å is obtained. This example demonstrates that MonoRes and ResMap should not be applied to maps sharpened with LocalDeblur, and in general to any map where noise suppression has been applied.

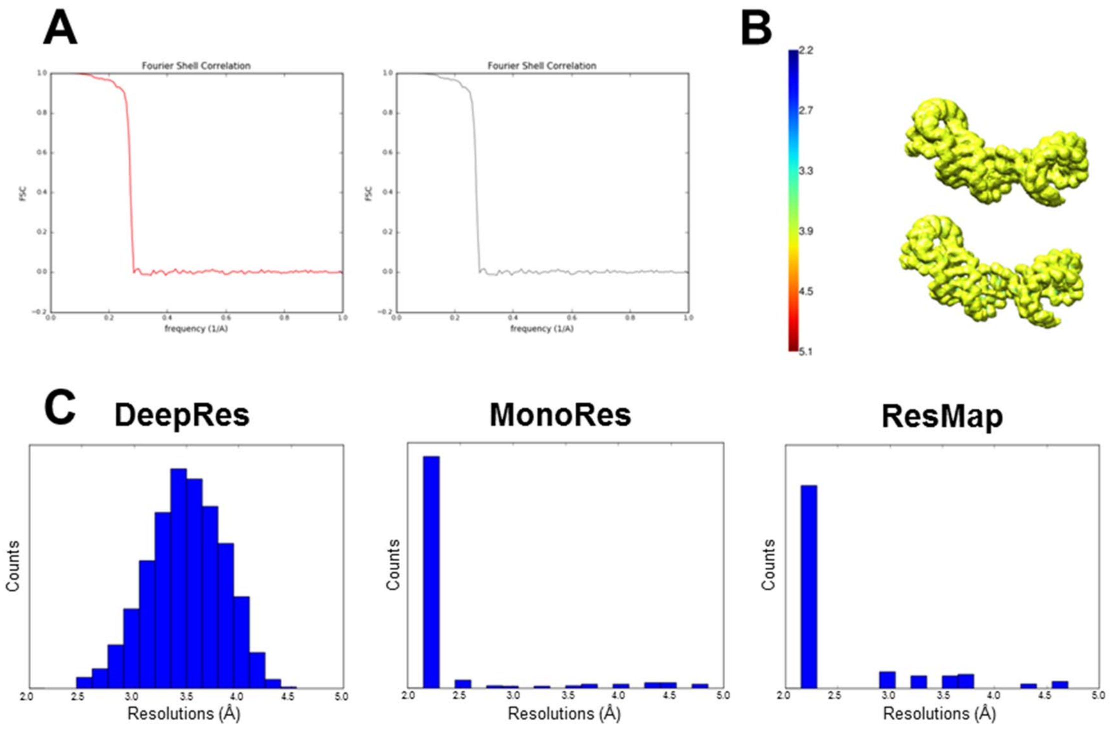

Figure S1 Some limitations of current methods. A) The FSC curve obtained for the simulated map of ϕ29 pRNA, with two Gausian noises added independently, is represented in red. The curve obtained once a B-factor of -60 is applied is represented in gray. No difference between both curves is detected. B) Resolution map obtained with MonoRes for the simulated map of ϕ29 pRNA, filtered at 4 Å, and added noise with mean 0 and 0.08 SD (above) and for the same map after applying a B-factor of -60 (below). In both cases the resolution map obtained is similar. C) The resolution histograms obtained by DeepRes, MonoRes and ResMap for the *E. coli* GroEL map sharpened with LocalDeblur.

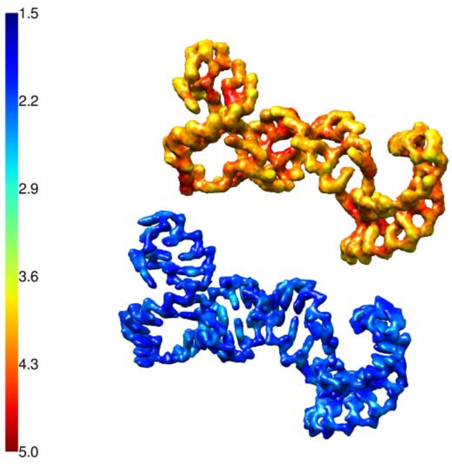

Figure S2 DeepRes resolution maps for simulated ϕ29 pRNA map. DeepRes result for the simulated map of ϕ29 pRNA (PDB: 3R4F) filtered at 2 Å (blue), and the same map after randomising the phases beyond 4 Å of resolution (orange).

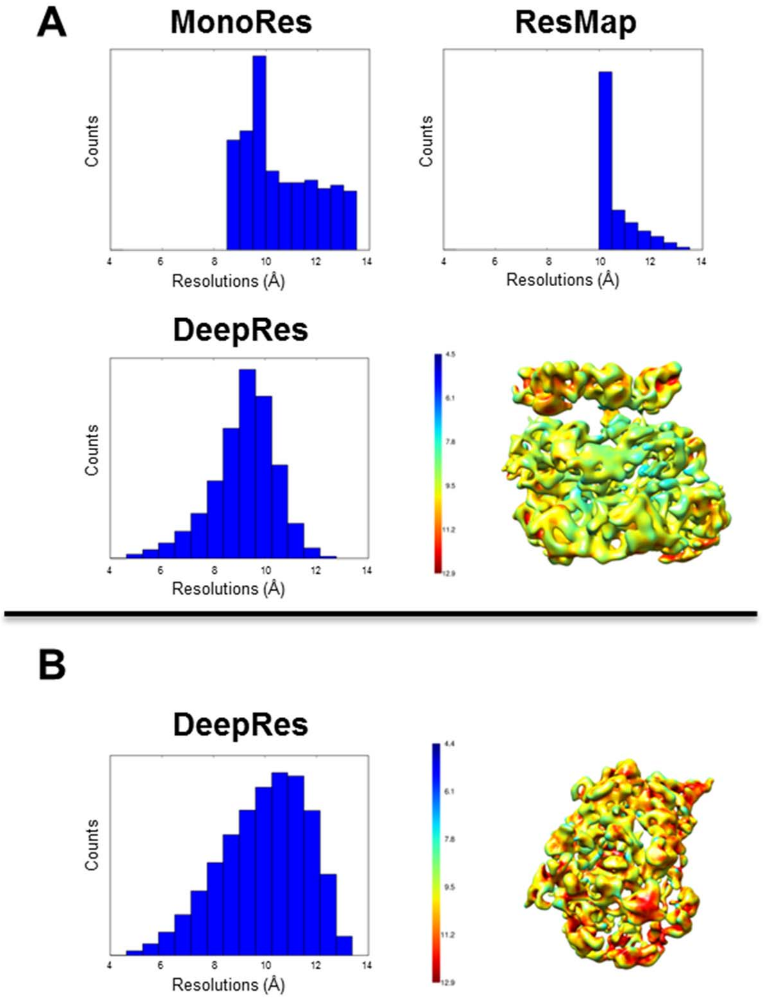

Figure S3 Local resolution results for experimental maps at medium resolution. A) The resolution histograms obtained by the different methods (MonoRes, ResMap and DeepRes) and the resolution map determined with DeepRes are shown for $HSP104_{DWB}$ (EMD-0376). B) The resolution histogram and the resolution map obtained by DeepRes are shown for the CMG helicase map (EMD-3320).

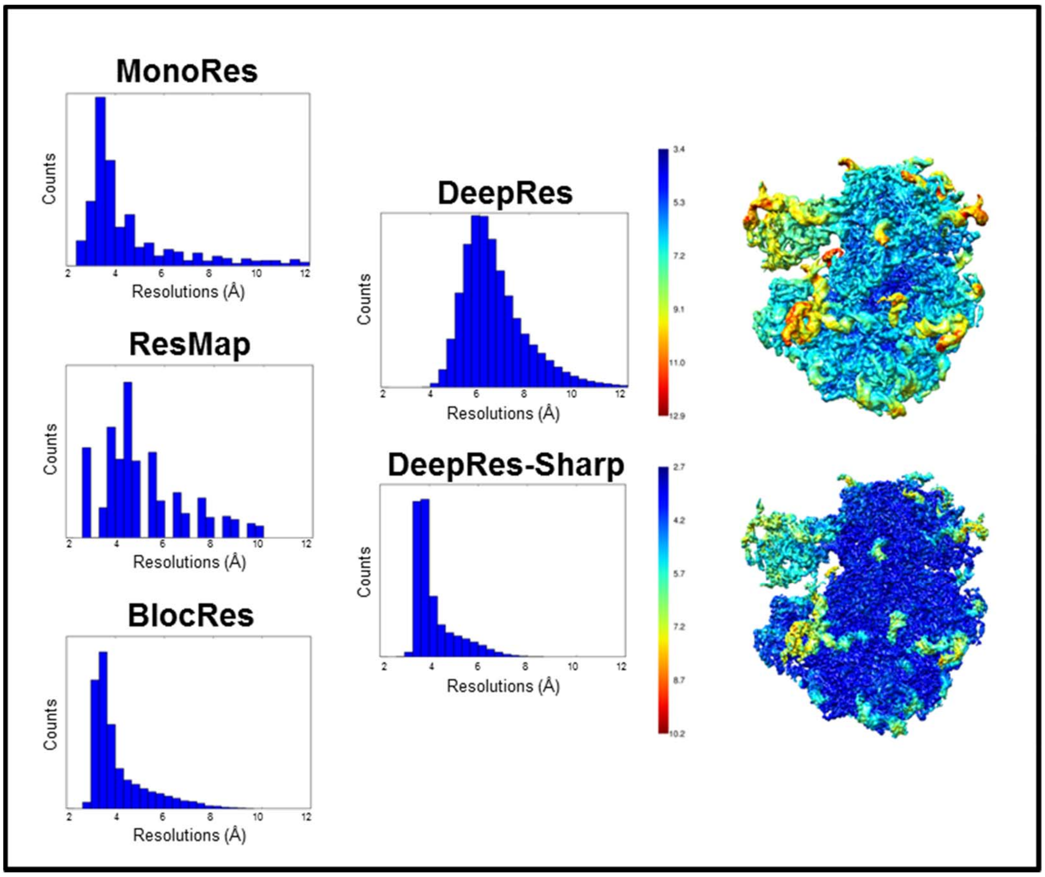

Figure S4 Changes in local resolution between unsharpened map and sharpened map detected with DeepRes. Histograms determined with MonoRes, ResMap y BlocRes for unsharpened map (left), DeepRes histograms for unsharpened and sharpened maps (center), and DeepRes resolution maps for unsharpened and sharpened maps (right) of *Plasmodium* 80S ribosome (EMD-2660). The sharpened map was obtained with AutoSharpen. Note that the color scales of the resolution maps have been modified for better visualization.

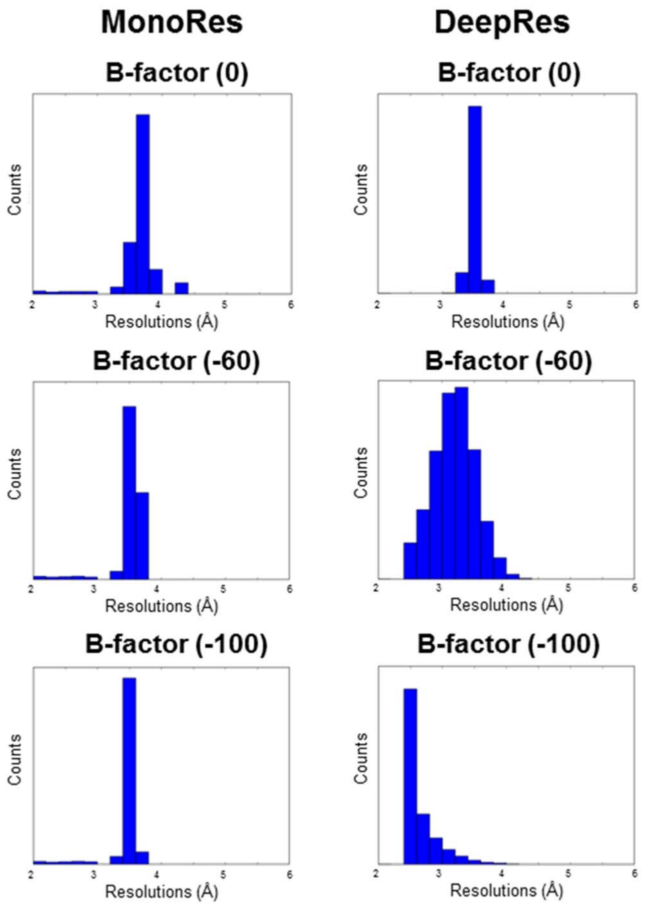

Figure S5 Comparison between Monores and DeepRes for simulated maps. The histograms obtained with MonoRes (left) and DeepRes (right) from the simulated map of the monomer 39-kDa human cartilage glycoprotein tetramer (PDB: 1HJV-chain A) filtered at 3.5 Å and sharpened with B-factor of -60 and -100.

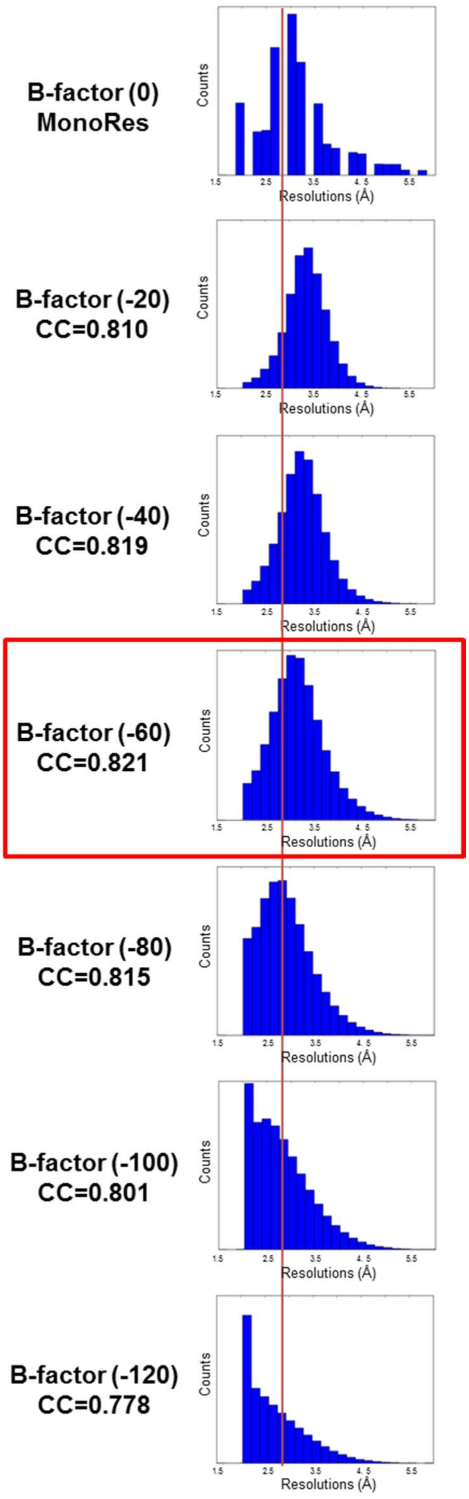

Figure S6 Over-sharpening detection. Comparison between MonoRes applied to the unsharpened map (top of the figure) and DeepRes applied to each B-factor sharpened map. The correlation (CC) of each sharpened map and the generated map from the deposited atomic model (PDB: ID-6BDF) were calculated using Phenix. The red line corresponds to the 20% percentile of the local resolution distribution of MonoRes.
